# Time Trends (2012–2020), Sex Differences and Predictors for Influenza Vaccination Uptake among Individuals with Chronic Obstructive Pulmonary Disease in Spain

**DOI:** 10.3390/jcm11051423

**Published:** 2022-03-04

**Authors:** Marta Fuentes-Alonso, Rodrigo Jimenez-Garcia, Ana Lopez-de-Andres, Jose J. Zamorano-Leon, David Carabantes-Alarcon, Isabel Jimenez-Trujillo, Sara Sanz-Rojo, Javier de Miguel-Diez

**Affiliations:** 1 Respiratory Department, Hospital General Universitario Gregorio Marañón, Instituto de Investigación Sanitaria Gregorio Marañón (IiSGM), Universidad Complutense de Madrid, 28040 Madrid, Spain; mfuentesa@salud.madrid.org (M.F.-A.); javier.miguel@salud.madrid.org (J.d.M.-D.); 2 Department of Public Health & Maternal and Child Health, Faculty of Medicine, Universidad Complutense de Madrid, IdISSC, 28040 Madrid, Spain; anailo04@ucm.es (A.L.-d.-A.); josejzam@ucm.es (J.J.Z.-L.); dcaraban@ucm.es (D.C.-A.); 3 Preventive Medicine and Public Health Teaching and Research Unit, Health Sciences Faculty, Universidad Rey Juan Carlos, Alcorcón, 28922 Madrid, Spain; isabel.jimenez@urjc.es; 4 Faculty of Health Science, Universidad Alfonso X El Sabio, Villanueva de la Cañada, 28691 Madrid, Spain; sara.sanz.rojo@gmail.com

**Keywords:** COPD, influenza, vaccine, sex differences, predictors, health survey, uptake, coverage

## Abstract

(1) Background: To analyze time trends, sex differences, and factors associated with influenza vaccination uptake among individuals with COPD in Spain, 2012–2020. (2) Methods: A cross-sectional study based on data from the European Health Surveys for Spain, 2020 (EHSS2020) and 2014 and from the Spanish National Health Interview Surveys for 2017 and 2012. (3) Results: The study included 65,447 participants. Prevalence of COPD was 5.9% (n = 3855). Overall, the influenza vaccination uptake among COPD patients was 57.8% versus 28.6% for those without COPD (*p* < 0.001). Men with COPD reported higher uptake than women in all the surveys studied. Neither the crude nor the multivariable analysis showed a significant variation change overtime for people with COPD. However, among those aged <65 years, crude uptake decreased from 2012 to 2020 (39.4% vs. 33.3%; *p* = 0.039). Over the entire period, men were vaccinated significantly more than women (OR 1.28; 95% CI 1.12–1.47). Among COPD participants, included in the EHSS2020, independent predictors of vaccine uptake included being male, higher age, reporting no current smoking and suffering cancer or heart disease. (4) Conclusions: In COPD patients, the influenza vaccination uptake is below desirable levels and did not improve from 2012 to 2020. Sex differences are found, with consistent and constant lower uptake among women with COPD. The observed lower uptake among COPD women and patients with unhealthy lifestyle requires increased attention.

## 1. Introduction

Chronic obstructive pulmonary disease (COPD) is characterized by the existence of persistent symptoms [1] and a chronic airflow limitation, fundamentally associated with tobacco use. It has a high prevalence and is one of the main causes of morbidity and mortality worldwide [2], causing a significant healthcare burden, both in primary and specialized care [3].

COPD exacerbations are a major public health problem. In fact, almost 60% of the global cost of COPD is attributable to such events [4]. Regardless of the economic impact that this entails, it is important to highlight the high clinical impact that exacerbations produce on the health status of patients [5], the progression of the disease and its vital prognosis [6].

Regarding the etiology of COPD exacerbations, at least 70% of them have an infectious origin, with respiratory viruses being the cause of approximately 30% of cases [7,8]. Among the viruses implicated in such exacerbations are influenza A and B viruses. In addition, we are currently experiencing the socioeconomic and health systems impact derived from the pandemic generated by SARS-CoV2 virus (COVID-19 disease). This virus can also trigger acute exacerbations in this group of patients. In this context, the risk of co-infections has special relevance, increasing the importance of adequate vaccination [9].

Influenza is an acute respiratory infectious disease of viral origin, with a high transmission capacity between people, which generates high economic and social costs as a consequence of its morbidity and mortality. Clinical practice guidelines recommend annual influenza vaccination for patients with COPD in order to reduce exacerbations related to this disease [10,11]. Despite the great efforts made by Public Health programs to immunize the population at risk, few countries have reached the vaccination target of 75% [12]. There are many difficulties in improving rates, including lack of social awareness, adequate healthcare resources, costs, undervaluation of the vaccine, and low access to them [13,14].

Among COPD patients, studies have also been carried out to find out their influenza vaccination uptake. Thus, for example, in the United Kingdom, figures of 36.1% have been reported in COPD patients older than 65 years compared to 23.8% in younger patients [15,16]. In other countries, such as Italy, influenza vaccine uptake in COPD patients was 30.5% during the period 2004–2005, rising to 74.8% in older people [17].

In Spain, in a study carried out by Santos Sancho et al. with data from the European Health Survey for Spain (EHS) of 2009, an influenza vaccination rate of 77.8% in COPD patients was evidenced, higher than the registered in other developed countries [18]. This fact could be related, among other things, to the free-of-charge vaccination program in Spain.

It has also been shown in our country that vaccination uptake of COPD patients increases above 65 years [19]. Similar results were described by Garrastazu et al., who also concluded that vaccinated COPD patients are an average of 6.2 years older than non-vaccinated patients [20].

Different studies have specifically analyzed the factors associated with a higher probability of influenza vaccination in people with COPD [21,22,23]. In many of them, it has been concluded that men with COPD are vaccinated more frequently than women with this disease, suggesting sex differences for this preventive intervention. In contrast, Vozoris et al. found a higher vaccination rate among women in Canada [24]. Further studies are required to clarify this issue. In any case, it is clearly demonstrated that patients with higher comorbidity tend to be vaccinated more often than those without associated diseases [25].

Most studies and reviews focused on patients with COPD show the need for new data on vaccination uptake that allow us to know the current compliance with the recommendations [26,27,28], as well as to deepen the study of the “non-compliant” and identify the associated factors [29,30]. This will provide us with very useful information to design appropriate strategies that can be applied in the design of new vaccination programs [31].

Based on the National health interview surveys conducted in Spain in years 2012, 2014, 2017 and 2020, we aimed to report trends in influenza vaccination uptake among individuals with COPD. We also assessed sex-differences and identified which sociodemographic and health-related variables were associated with uptake among this high-risk population.

## 2. Materials and Methods

### 2.1. Design and Study Population

We performed a cross-sectional study based on data from the European Health Surveys for Spain conducted in years 2020 (EHSS2020) and 2014 (EHSS2020) and the Spanish National Health Interview Surveys for years 2017 (SNHIS2017) and 2012 (SNHIS2012). The data collection period for these surveys is 52 weeks. Details on these surveys, including the methodology, questionnaires, and non-response rates, can be found elsewhere [32,33,34,35].

Briefly, to obtain national representative estimations, these surveys use three-stage sampling. The first stage is the census sections; the second stage, family dwelling; and in the final stage, an adult aged 15 years or over is randomly selected for each family [31,32,33,34]. Computer-assisted home-based personal interviews were used over time for data collection in all surveys. However, in the EHSS2020, due to the COVID-19 pandemic, from weeks 35 to 52 (once the lockdown period started in Spain), data were collected using computer-assisted personal telephone interviews [32].

The study population comprised all persons aged ≥40 years. The reason for this cut point is the very low prevalence of COPD in Spain under this age [36].

### 2.2. Study Variables

Study variables were created using identical questions in all the national surveys included in our investigation [32,33,34,35].

The primary study variable was the uptake of the influenza vaccine. We considered vaccinated those participants who answered affirmatively to the question “Were you vaccinated against influenza during the last vaccination campaign?” Those who answered “don’t know” or refused to reply were excluded from the study population.

The presence of self-reported COPD was collected with the question, “Have you been diagnosed by a physician with COPD?”.

As independent variables, we analyzed socio-demographic variables, such as sex, age, marital status, educational level and social class. Lifestyle variables included obesity, currently smoking, alcohol consumption and physical activity.

Health-related variables analyzed were self-rated health, self-reported chronic conditions for which annual influenza vaccination is recommended in Spain, regardless of the age group (heart disease, cancer, cerebrovascular diseases, diabetes and renal disease) and mental disease (depression and/or anxiety).

Detailed descriptions of the questions of the EHSS and SNHIS questionnaires used, and categories applied to create the above-mentioned variables, are shown in Table S1.

The presence of any of the mentioned chronic medical conditions was also analyzed as a dichotomous variable, “any chronic conditions”.

### 2.3. Statistical Analysis

We described and compared vaccination uptake according to age group and sex between those with and without COPD for the four national health surveys. Differences according to COPD status were analyzed using the chi-square test. To assess the time trend from 2012 to 2020 in the vaccination uptake among those with and without COPD, we constructed logistic regression models. Uptake in SNHIS2012 was used as the reference category.

Using data from the EHSS2020 survey, we compared the vaccination uptake between the COPD population and those without this condition stratified by study variables. Uptake is expressed as a percentage with the 95% confidence interval (CI). Differences according to study variables were analyzed using the chi-square test.

Additionally, an unconditional multivariable model was constructed to identify which study variables were associated with vaccination uptake among those participants with COPD included in the EHSS2020.

The models multivariable were constructed based on the following strategy: (i) univariable analysis of each variable; (ii) selection of the variables to be included in the multivariable analysis, which included all variables with a significant association (*p* < 0.10) in the univariable analysis and those identified as important in the literature search; (iii) verification of the importance of each variable included in the model using the Wald statistic and the comparison of the successive models with the previous models using the likelihood ratio test; (iv) possible linearity between variables analyzed and interactions were determined after the model was constructed; and (v) the Hosmer–Lemeshow test was used to assess the goodness of fit for the regression models constructed. The association estimates were expressed as the odds ratio (OR) with its 95% CI.

The statistical analysis was performed using SPSS 25.0 (IBM Corp, Armonk, NY, USA), with a *p* value < 0.05 considered statistically significant (two-tailed).

### 2.4. Ethical Aspects

The databases of the EHSS2020, EHSS2014, SNHIS2017 and 2012 SNHIS2012 used in this investigation were freely downloaded from the Spanish Ministry of Health webpage [31,32,33,34]. All downloaded data are completely anonymous, and informed consent was obtained from participants prior to the interview. Therefore, and according to Spanish legislation, the approval of an ethics committee was waived.

## 3. Results

### 3.1. Influenza Vaccination Uptake in the COPD Population from 2012 to 2020

The study population included 65,447 participants aged 40 years or over, interviewed in the four surveys analyzed. The total prevalence of self-reported physician-diagnosed COPD was 5.9% (n = 3855), and this prevalence remained stable overtime. The influenza vaccination uptake among COPD subjects was 57.8% (95% CI 56.2–59.4) with 28.6% (95% CI 28.2–29.0) for those not suffering this condition (*p* < 0.001).

**Figure 1:**
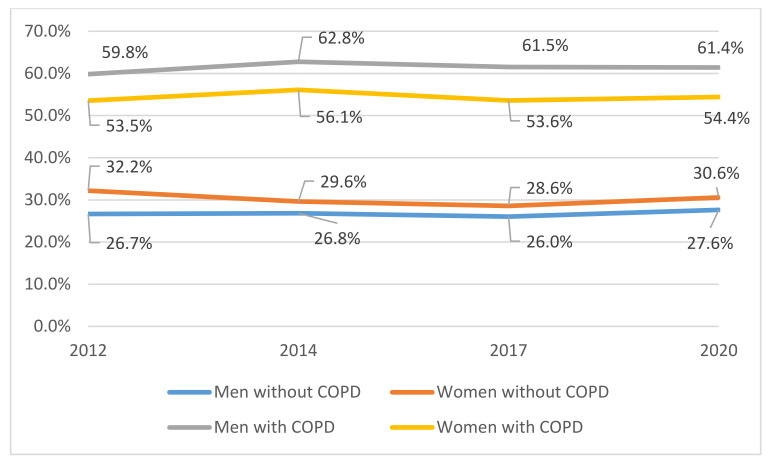
Time trend from 2012 to 2020 of influenza vaccination uptake, according to sex and COPD status.

Shown in Figure 1 is the time trend in vaccination uptake according to sex. Men with COPD reported higher uptake than women in all the surveys studied. Over the entire period, the values were 61.4% (95% CI 58.5–62.9) for men versus 55.0% (95% CI 52.8–57.2) for women (*p* < 0.001). The uptake did not show a significant variation over time for neither men nor women with COPD. Unlike in the COPD population, the total uptake from 2012 to 2020 among non-COPD women was significantly higher than among non-COPD men (30.2% vs. 26.8%; *p* < 0.001).

**Figure 2:**
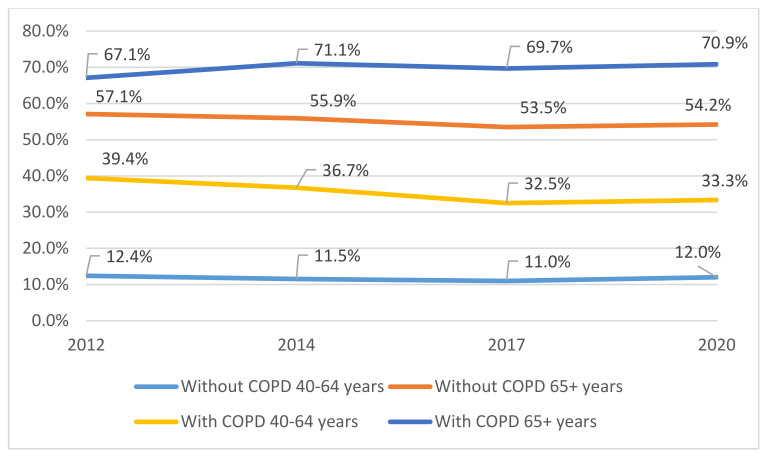
Time trend from 2012 to 2020 of influenza vaccination uptake, according to age groups and COPD status.

In Figure 2 appears the time trend in the uptake among participants with and without COPD according to age groups. For both populations, the uptake was much higher in the 65 and over years’ group, compared with those aged 40 to 64 years. Among COPD subjects, uptake reached the highest value in 2020 (70.9%) for those aged ≥65 years, with a slight, but not significant, improvement from year 2012 (67.1%). However, among those aged 40 to 64 years, the uptake was 33.3% in 2020 with a significant reduction from 2012 (39.4%; *p* = 0.039).

The results of the multivariable logistic regression model to assess the age–sex adjusted time trend are shown in Table 1. Among those with self-reported COPD, no significant change in uptake was found over time. Men were vaccinated significantly more than women (OR 1.28; 95% CI 1.12–1.47), and the probability of receiving the vaccine rose with age. Compared with the reference category, 40–64 years’ group, we found an OR of 3.24 (95% CI 2.73–3.85) for the 65–74 years’ group and 4.97 (95% CI 4.23–5.83) for those over 74 years.

For the non-COPD population, women received the vaccine significantly more than men (OR1.09; 95% CI 1.05–1.13) and uptake increased with age. Among those without COPD, a significant decrease was observed from 2012 to 2020 after multivariable adjustment (Table 1).

### 3.2. Influenza Vaccine Uptake among COPD Participants in the EHSS2020 According to Study Variables

Table 2 shows influenza vaccination uptake, according to study variables among participants with COPD interviewed in the EHSS2020.

The results revealed that uptake reached 74.5% in those aged ≥75 years, and the proportion of vaccinated increased significantly with age. Uptake was significantly associated with sex (61.4% for men vs. 54.4% for women; *p* = 0.040) and educational level (66.3% for those primary school or less vs. 48.8% with secondary school or equivalent vs. 46.0% with higher education; *p* < 0.001).

Those who did not smoke or did not consume alcohol had significantly higher uptake than those with these unhealthy lifestyles (63.8% vs. 39.0%; *p* < 0.001 and 61.0% vs. 52.7%; *p* = 0.017, respectively).

Table 2 also shows the influenza vaccination uptake rates among participants with the chronic diseases for which the influenza vaccine is recommended. The highest vaccination rate was found in those with cancer (69.9%), followed by heart disease (65.1%) and diabetes (62.8%). For the first two conditions, the uptake was significantly higher among participants with COPD who reported to also suffer them, compared to those without. The lowest rates were observed for those with COPD and concomitant mental diseases (54.7%).

Suffering any of the chronic conditions increased uptake (59.9% vs. 53.3), but the difference was not significant (*p* = 0.059).

### 3.3. Multivariable Analysis to Identify Variables Independently Associated with Influenza Vaccine Uptake among COPD Participants in the EHSS2020

The results of the logistic regression models to identify variables associated with vaccination are shown in Table 3. Being a man was identified as a positive predictor of influenza vaccination uptake (OR 1.336; 95% CI 1.022–2.199). The probability of being vaccinated also increased with age (OR 3.844 for the 65–74-year group and OR 5.855 for those aged 75 or over when compared to those aged 40 to 64 years).

Finally, among COPD participants, those reporting no current smoking (OR 1.508; 95% CI 1.041–2.183), suffering heart disease (OR 1.352; 95% CI 1.039–1.994) or cancer (OR 1.273; 95% CI 1.006–1.768) were more likely to be vaccinated.

## 4. Discussion

The present study showed that COPD patients have an influenza vaccination rate below desirable levels (57.8%), although this figure was higher than that of those who do not suffer from this disease (28.6%). For both populations, adherence to the vaccine was higher in the group of patients aged 65 years or older, compared to those of a younger age. In fact, among subjects with COPD, vaccination reached a value of 70.9% in 2020 for people aged ≥65 years. However, among people aged 40 to 64 years, the rate was 33.3% in the same year, with a significant reduction compared to data registered in 2012 (39.4%). Another interesting finding from our study was that women with COPD received the vaccine less frequently than men (55% vs. 61.4%). However, in the non-COPD population, the inverse association was found (30.2% vs. 26.8%). Regarding other variables, our study showed that, in COPD patients who did not smoke or did not consume alcohol, vaccination was significantly higher than in those with unhealthy lifestyles (63.8% vs. 39% and 61% vs. 52.7%, respectively). Finally, the highest vaccination rate was found in COPD participants with concomitant cancer (69.9%) followed by those with heart disease (65.1%) and diabetes (62.8%).

Our results are like those found in other studies. Despite evidence-based recommendations, only 50% to 60% of COPD patients are vaccinated [20,37]. For example, in a study carried out by Eagan et al. [38] in Norway, the overall prevalence of influenza vaccination in COPD patients was 59%. Although this prevalence is suboptimal, like that obtained in our study, it is higher than that registered in other, older studies also carried out in developed countries [39,40]. Thus, in a study conducted in four European countries, based on telephone surveys, and published in 2003, influenza vaccination rates in COPD patients were 8% in Poland, 11% in Sweden, 20% in Germany and 30% in Spain [31].

Regarding gender, both in our study and in other previously published [22], the data show that men with COPD are vaccinated in a higher proportion than women. In the same way, in a recent study carried out by Ruiz Azcona et al., vaccination rates of 62.6% were achieved in men compared to 53.6% in women [41]. In some cases, the figures were even doubled, as it was evidenced in a study carried out in Spain in 2009 with data obtained from the European Health Survey (EHS) [18].

A remarkable result of our investigation is that, in contrast to the COPD population, total vaccination uptake among non-COPD women was significantly higher than among non-COPD men. Higher uptake by women than by men in the general population was reported in many, but not all, investigations conducted in Spain [42,43,44,45,46,47]. In our country, previous studies showed that uptake may be mediated by age, with better rates for males among those aged ≥65 years and for females among those under this age [45]. The very high mean age of the COPD population (total 69.58 years; SD 12.52, for women 69.61 SD 13.34 years vs. 69.54 SD 12.52 years for men *p* = 0.125) compared with the non-COPD population (total 61.71 years; SD 14.10, for women 63.02 SD 14.53 years vs. 60.43 SD 13.44 years for men *p* < 0.001) could partly explain this surprising association. Another possible reason is that in Spain, concomitant cardiovascular disorders are significantly more frequent in male COPD patients, and therefore they receive more recommendations for vaccination than women. Jenkins et al. reported that sex bias exists among COPD patients, suggesting that women receive later diagnosis and worse treatment than men [48]. As suggested by other authors, this might also result in sex differences in the use of preventive healthcare services and visits to physicians, contributing to lower vaccine uptake, although this hypothesis warrants further investigation [46,47,49].

Regarding the age of vaccination, most of the studies carried out in Spain [42,43,44,45,46,47,50,51] and in other countries, such as the United States, Canada, Germany, and other European regions [24,52], agree that in individuals older than 64 years, the probability of vaccination is higher than in the younger groups [30,50]. So, in the study carried out by Saeed et al., a vaccination rate of only 44.8% was achieved in COPD patients aged between 40 and 64 years [53].

Some authors suggest that this could be justified by the fact that 65 years is the age from which vaccine recommendation becomes universal [42,43,50,51]. On the other hand, it is possible that younger patients are vaccinated less frequently since they are usually affected by the disease in a milder way and usually have a more recent diagnosis [19]. It has been suggested that vaccination strategies based on age groups are more effective than those directed at high-risk groups [54].

In our study, we also observed higher vaccination uptake in COPD patients who did not smoke or did not consume alcohol. These findings are consistent with the results found in previous studies [22,41]. In the study by Garrastazu et al., 80% of non-smokers were vaccinated, and the group of ex-smokers were vaccinated to a lower proportion (63.9%) [20]. This finding could be because patients who have stopped smoking could be those affected with a more severe COPD and with a greater fear of influenza infection [18].

Regarding alcohol consumption, in an analysis of data from COPD patients previously included in an epidemiological study carried out in primary care in Spain, a vaccination uptake for influenza of 89.9% was observed in patients who did not have this habit. On the other hand, those who presented a concomitant chronic disease, such as diabetes mellitus and/or heart disease, were vaccinated in a significantly higher percentage (92.4% versus 80.3%) than those who did not suffer from these pathologies [19]. These data are in line with those obtained in our study and reflect the existence of higher vaccination uptake in patients who perceive that they have a worse health.

A surprising finding of our investigation was the higher vaccine uptake among people with COPD with lower education and social class in the univariate analysis, even if these associations become insignificant after the multivariable adjustment. A meta-analysis to systematically appraise and quantify social factors associated with influenza vaccine uptake amongst individuals aged 60 years in Europe concluded that uptake was associated with higher income (OR = 1.26; 95% CI: 1.08–1.47) and higher education (OR = 1.05 (95% CI: 1–1.11) [55]. Lucyk et al. explained that the direction of the association (positive and negative) is not always clear and seems to vary depending on how socioeconomic status is measured, as a single measure or as a combination of educational level, income and social class [56].

In Spain, previous studies found either no association, or that lower education, social class, or monthly income are positive predictors for vaccination [45,47,57,58,59].

Dios-Guerra C et al. found that after multivariable analysis, among Spaniards aged 65 year or over, people of low social classes were 1.24 (95% CI 1.13–1.34; *p* < 0.001) times more likely to uptake vaccination than those of high social classes. Furthermore, those without any studies were 1.15 (95% CI 1.04–1.27; *p* = 0.006) times more likely to participate in vaccination campaigns than people with a higher educational level (primary, secondary or university education) [47].

It has been suggested that this may be due to people from higher social classes or higher educational level being possibly more susceptible to both anti-vaccination campaigns and the increased perception of the potential risks of vaccinations [55]. Further studies are needed to clarify theses associations.

The prevalence of comorbidities has increased over time among men and women with COPD [18,22]. Specifically, the prevalence of heart disease has increased from 24.78% in 2012 to 28.02% in 2020. However, the increment has been different according to sex, from 23.39% to 26.10% among women with COPD (12.1% increase), and from 25.89% to 30.04% among men with COPD (16.03% increase). The time trends in the comorbid conditions may have contributed to the evolution of vaccine uptake overtime.

Our results reinforce the need to carry out campaigns aimed at society and health personnel to promote the recommendations arising from the analysis of national and international data. In this sense, Blank et al. demonstrated that the most important factor motivating patients to get vaccinated is professional recommendation [60]. Among the various vaccination strategies with proven effectiveness are telephone calls, personal letters or emails, the design of vaccination schedules, the identification of high-risk patients through databases, and vaccination educational programs [61,62]. These strategies should be considered and implemented in our country.

With the arrival of the new disease caused by SARS-CoV-2 (COVID-19), it is even more necessary to identify patients at high risk of complications. The evidence available to date suggests a higher incidence, severity, and mortality of COVID-19 in individuals with COPD [53]. Although the direct effect that influenza vaccination could have on patients with COVID-19 remains uncertain, subjects with COPD are at high risk of coinfection, and now more than ever, it is essential to achieve adequate vaccination rates in this group of patients.

The main strength of this study is the use of a large nationally representative sample, which provides greater external validity to our results since we have a sample size of more than 65,000 patients. Furthermore, unlike most studies that cover a single season, our study covered several influenza vaccinations seasons, specifically the years 2012, 2014, 2017, and 2020. Furthermore, we analyzed variables that are not usually collected in medical records.

However, our study has several limitations. First, the validity of self-reported influenza vaccination has not been evaluated in the Spanish surveys. However, in previous studies, self-reported influenza vaccination was assessed and considered adequate, providing a very high sensitivity and a moderate–high specificity [63,64,65]. Regarding the validity of self-reported COPD diagnosis, we also lack a validation study. Furthermore, we ignore whether or not sex differences exist in the validity of self-reported COPD, which could affect our results. In any case, we do not have any indication that suggests that COPD women are less likely to have true COPD. However, women more frequently than men suffer the asthma–COPD overlap syndrome, and this could result in misclassification [66]. This issue requires further investigation. Secondly, it is very likely that the patients, in addition to the influenza vaccination, received the pneumococcal vaccine at some point, which could act as a confounding factor, so it would be interesting to obtain these data for each of our patients in future surveys. Thirdly, it is important to consider the influence that the current pandemic situation may have had on the flu vaccination campaign for the 2020 season. Difficulty in accessing health systems may have affected vaccine uptake. However, in Spain, influenza vaccine programs start in October and finish by February, so the effect of the COVID-19 pandemic, if any, would be of small magnitude. Fourthly, on the other hand, the information obtained by the patients could be conditioned by the desire to provide socially acceptable responses and altered by memory biases. Fifthly, unfortunately, the SNHS only collects information on current smoking but not on smoking history, so the influence of this factor could not be assessed. Previous investigations found that statistically significant sex-related differences according to smoking status showed a higher percentage of men as compared with women in the groups of current smokers and ex-smokers. On the other hand, never-smokers were more frequent in women (9.1%) than in men (0.6%), and women had lower pack–years [66,67]. Finally, the initial non-response rate for the surveys used range between 30% and 40%, so the existence of a non-response bias must be considered [32,33,34,35].

## 5. Conclusions

In conclusion, the influenza vaccination uptake is below desirable levels in COPD patients and did not improve from 2012 to 2020. Current results suggest a worsening of uptake among younger COPD individuals. Sex differences were found, with consistent and constant lower uptake among women with COPD. COPD participants with healthy lifestyles and associated comorbidities showed higher influenza vaccination. These results reflect the need to continue studying new strategies to improve uptake, especially among women, younger patients, and those with unhealthy lifestyles.

## Figures and Tables

**Table 1:** Multivariable logistic regression to assess time trend from 2012 to 2020 of influenza vaccination uptake, according to COPD status.

COPD	Variable	Categories	Odds Ratio	95% Confidence Interval	
Yes	Sex	Women	Ref		
		Men	1.283	1.119	1.470
	Age groups	40–64 years	Ref	-	-
		65–74 years	3.243	2.734	3.848
		75 years and over	4.968	4.232	5.831
	Year of survey	2012	Ref	-	-
		2014	1.075	0.888	1.301
		2017	0.973	0.808	1.173
		2020	1.022	0.839	1.245
No	Sex	Men	Ref	-	-
		Women	1.088	1.045	1.133
	Age groups	40–64 years	Ref	-	-
		65–74 years	6.267	5.971	6.577
		75 years and over	13.733	13.079	14.420
	Year of survey	2012	Ref	-	-
		2014	0.944	0.890	1.000
		2017	0.863	0.815	0.915
		2020	0.914	0.863	0.968

Ref: Reference category.

**Table 2:** Influenza vaccine uptake among COPD participants in the EHSS2020 according to study variables.

Variable	Categories	%	95% CI	*p*
Age groups	40–64 years	33.3	30.1–36.5	<0.001
	65–74 years	65.8	59.6–71.9	
	75 years and over	74.5	69.7–79.3	
Sex	Man	61.4	56.6–66.2	0.040
	Women	54.4	49.8–59.0	
Marital status	Married	55.6	50.8–60.3	0.117
	Not married	59.9	55.2–64.6	
Education level	Primary school or less	66.3	61.9–70.7	<0.001
	Secondary school or equivalent	48.8	43.1–54.5	
	Higher education	46.0	36.2–55.8	
Social class	High	50.6	40.0–61.2	0.205
	Medium	59.8	53.9–65.8	
	Low	57.9	53.4–62.4	
Self-rated health	Very good/good	53.1	46.8–59.5	0.089
	Fair/poor/very poor	59.6	55.6–63.5	
Obesity	No	57.4	53.3–61.5	0.581
	Yes	56.8	50.3–63.4	
Current smoking	No	63.8	60.1–67.6	<0.001
	Yes	39.0	32.3–45.7	
Alcohol consumption	No	61.0	56.7–65.2	0.017
	Yes	52.7	47.3–58.0	
Physical activity	No	56.4	51.9–60.8	0.366
	Yes	59.5	54.5–64.6	
Heart disease	No	54.9	50.9–58.8	0.007
	Yes	65.1	59.0–71.2	
Cancer	No	56.0	52.5–59.6	0.008
	Yes	69.9	61.0–78.8	
Cerebrovascular diseases	No	57.8	54.3–61.2	0.896
	Yes	56.9	43.3–70.5	
Diabetes	No	56.3	52.5–60.1	0.114
	Yes	62.8	55.8–69.8	
Renal disease	No	57.8	54.3–61.4	0.899
	Yes	57.1	47.3–66.9	
Mental disease	No	59.5	55.3–63.7	0.173
	Yes	54.7	49.3–60.2	
Any chronic condition	No	53.0	47.0–59.0	0.059
	Yes	59.9	55.9–63.9	

**Table 3:** Multivariable logistic regression to assess variables associated with influenza vaccination uptake among participant in the EHSS2020 with COPD.

Variable	Categories	Odds Ratio	95% Confidence Interval	
Sex	Women	Ref		
	Men	1.336	1.022	2.199
Age groups	40–64 years	Ref	-	-
	65–74 years	3.844	2.667	5.541
	75 years and over	5.855	4.126	8.309
Current smoking	Yes	Ref	-	-
	No	1.508	1.041	2.183
Heart disease	No	Ref	-	-
	Yes	1.352	1.039	1.994
Cancer	No	Ref	-	-
	Yes	1.273	1.006	1.768

Ref: Reference category.

## Data Availability

According to the contract signed with the Spanish Ministry of Health and Social Services, which provided access to the databases from Spanish National Health Survey and European Health Survey for Spain, we cannot share the databases with any other investigator, and we have to destroy the databases once the investigation has concluded. Consequently, we cannot upload the databases to any public repository. However, any investigator can apply for access to the databases by filling out the questionnaire available at http://www.msssi.gob.es/estadEstudios/estadisticas/estadisticas/estMinisterio/SolicitudSNHSdocs/Formulario_Peticion_Datos_SNHS.pdf (accessed on 4 January 2022). All other relevant data are included in the paper.

## Supplementary Materials

The following supporting information can be downloaded at: https://www.mdpi.com/article/10.3390/jcm11051423/s1, Table S1: Definition of study variables used in our investigation according to the questions included in the European Health Survey for Spain 2020 and 2014 and the Spanish National Health Interview Surveys for years 2017 and 2012.
