# Supplementary material for: Time Trends (2012–2020), Sex Differences and Predictors for Influenza Vaccination Uptake among Individuals with Chronic Obstructive Pulmonary Disease in Spain
Source: J Clin Med. 2022 Mar 4;11(5):1423. doi: 10.3390/jcm11051423 (PMC8910978; doi:10.3390/jcm11051423)
Supplement: Supplementary file 1 [file jcm-11-01423-s001.zip › jcm-1566031-supplementary.pdf]

Table S1. Definition of study variables used in our investigation according to the questions included in the European Health Survey for Spain 2020 and 2014 and the Spanish National Health Interview Surveys for years 2017 and 2012.

QUESTIONS	DESCRIPTION AND ANSWER	VARIABLE NAME	CATEGORIES
Were you vaccinated against influenza during the last vaccination campaign?	Those that answered "Yes" were considered vaccinated.	Influenza vaccination	No<br>Yes
Which is your sex?	Man<br>Woman	Sex	Man<br>Woman
How old are you?	Age in years	Age groups	45-64<br>65 or older<br><br>40-64<br>65-74<br>75 and over
What is your marital status?	1. Single<br>2. Married<br>3. Widower<br>4. Separated<br>5. Divorced	Marital status	1. Married: Option 2<br>2. Not married: options 1, 3, 4 and 5
What level of education have you completed?	1. Does not know how to read or write<br>2. Incomplete primary education<br>3. Complete primary education<br>4. First stage of Secondary Education, with or without a qualification<br>5. Elementary Spanish Upper Secondary Education<br>6. Upper secondary education<br>7. Intermediate vocational training or equivalent<br>8. Advanced vocational training or equivalent<br>9. University studies or equivalent<br>10. Over university (master, PhD….)	Educational level	1. Primary school or less: Options 1 to 3<br>2. Secondary school or equivalent: Options 4 to 8<br>3. Higher education: Options 9 and 10

The social class categories have been taken from the proposal made Spanish Society of Epidemiology’s (SEE) Working Group on Determi which social class is assigned according to occupation.	National Classification of Occupations 2011 (CNO2011)	Social Class	1. High<br>2. Medium<br>3. Low
In the past twelve month, how is your perception of your general he status?	1. Very good<br>2. Good<br>3. Fair<br>4. Bad<br>5. Very bad	Self-rated health	1. Very good/good: Options 1 and 2<br>2. Fair/poor/very poor: Options 3 to 5
1. Could you tell me how tall you are, approximately, without shoes?<br>2. Could you tell me your weight, approximately, without shoes and	Body mass index is calculated with the formulae: Weight in kg/ (Height in meters)^2	Obesity	1. Yes: If body mass index is 30 or over<br>2. No: Yes: If body mass index is under 30
During the past 12 months, how often have you had alcoholic bever any kind (i.e. beer, wine, spirits, distilled and mixed drinks, or other beverages)?	1. Daily or almost daily<br>2. 5-6 days per week<br>3. 3-4 days per week<br>4. 1-2 days per week<br>5. 2-3 days in a month<br>6. Once a month<br>7. Less than once a month<br>8. Not in the last 12 months, have I stopped drinking<br>9. Never or just a few sips to taste it throughout life	Alcohol consumption in last 12 months	1. Yes: Options 1 to 6<br>2. No: Option 7 to 9
Could you tell me if you smoke?	1. Yes, I smoke daily<br>2. Yes, I smoke, but not daily<br>3. I don't currently smoke but have smoked before<br>4. I neither smoke nor have I ever smoked regularly	Current smoking	1. Yes: Options 1 and 2<br>2. No: Options 3 and 4

Which of these possibilities best describes how often you do some [illegible] activity in your free time?	1. I don't exercise. I occupy my free time almost completely sedentary<br>2. I do some occasional physical or sports activity<br>3. I do physical activity several times a month<br>4. I do sports or physical training several times a week	Physical activity	1. No: Option 1<br>2. Yes: Option 2 to 4
#1.*Do you have, or have you ever had any of the following diseases or medical conditions?*<br>Those who answered "yes" completed question #2<br><br>#2.*Have you suffered from that disease/medical condition over the last 12 months?*<br>Those who answered "yes" completed question #3<br><br>#3. *Have you been diagnosed by a physician with this disease/health condition?*	A list of 32 conditions was given to the interviewee including: myocardial infarction, angina pectoris, coronary diseases and other hear disease.	Heart disease	Yes: when answered affirmatively to the question #3 *for* any of the listed chronic conditions.<br><br>No: any other given answer
	A list of 32 conditions was given to the interviewee including: Malignant Tumors	Cancer	Yes: when answered affirmatively to the question #3 *for* Malignant Tumors<br><br>No: any other given answer.
	A list of 32 conditions was given to the interviewee including: stroke, cerebral infarction, brain embolism, brain hemorrhage.	Cerebrovascular diseases	Yes: when answered affirmatively to the question #3 *for* any of the listed chronic conditions<br><br>No: any other given answer.
	A list of 32 conditions was given to the interviewee including Diabetes Mellitus.	Diabetes Mellitus	Yes: when answered affirmatively to the question #3 *for* diabetes mellitus<br><br>No: any other given answer.
	A list of 32 conditions was given to the interviewee including Renal disease.	Renal disease	Yes: when answered affirmatively to the question #3 *for* Renal disease<br><br>No: any other given answer.

	A list of 32 conditions was given to the interviewee including: depression and anxiety	Mental disease	Yes: when answered affirmatively to the question #3 *for* any of the listed chronic conditions<br><br>No: any other given answer.
As described in the five previous variables	As described in the previous variables	Any chronic condition	Yes (one or more of the following chronic conditions listed above<br>No Those without any of the chronic conditions listed above
